# A Novel Method for Multi-Fault Feature Extraction of a Gearbox under Strong Background Noise

**DOI:** 10.3390/e20010010

**Published:** 2017-12-26

**Authors:** Zhijian Wang, Junyuan Wang, Zhifang Zhao, Rijun Wang

**Affiliations:** School of Mechanical and Power Engineering, North University of China, Xueyuan Road, Taiyuan 030051, China

**Keywords:** minimum entropy deconvolution, multipoint optimal minimum entropy deconvolution adjusted, signal ratios, multiple faults

## Abstract

Strong background noise and complicated interfering signatures when implementing vibration-based monitoring make it difficult to extract the weak diagnostic features due to incipient faults in a multistage gearbox. This can be more challenging when multiple faults coexist. This paper proposes an effective approach to extract multi-fault features of a wind turbine gearbox based on an integration of minimum entropy deconvolution (MED) and multipoint optimal minimum entropy deconvolution adjusted (MOMEDA). By using simulated periodic transient signals with different noise to signal ratios (SNR), it evaluates the outstanding performance of MED in noise suppression and reveals the deficient in extract multiple impulses. On the other hand, MOMEDA can performs better in extracting multiple pulses but not robust to noise influences. To compromise the merits of them, therefore the diagnostic approach is formalized by extracting the multiple weak features with MOMEDA based on the MED denoised signals. Experimental verification based on vibrations from a wind turbine gearbox test bed shows that the approach allows successful identification of multiple faults occurring simultaneously on the shaft and bearing in the high speed transmission stage of the gearbox.

## 1. Introduction

Wind turbines are important in modern industrial electric power production. The health status of the gearbox directly affects the working condition of the wind turbine system. In the event of failure, it will be costly, therefore, its fault diagnosis technology is highly valued. If the position of faults can be predicted accurately, they can be effectively avoided. Research into new multi-fault diagnosis methods plays an important role in ensuring reliable gearbox performance. When faults occur in the gears and inner race, outer race or rolling elements of the bearings, the transmission system will be affected, and periodic pulses appear in the vibration signals [1,2,3]. It is usually difficult to diagnose potential faults, especially when multiple faults exist under strong background noise, vibrations excited by several faults are combined with each other non-linearly and non-stationarily. Thus, simultaneous detection of multiple faults is still a big challenge in the monitoring and diagnosis of rotating machinery [4,5,6]. In fact, the vibration signal feature extraction aims to extract the shock component, or in other words, to find an optimal filter to reduce the shock of noise on the fault signal. Minimum entropy deconvolution (MED), which is an adaptive system identification method, was first proposed by Wiggins [7]. The basic principle of MED is to solve the deconvolution results to highlight a few large spikes, which is a necessary prerequisite for minimum entropy deconvolution. The maximum kurtosis is used as the iteration termination condition [8]. According to the maximum principle of kurtosis, a larger kurtosis value indicates a larger proportion of the shock component in the signal. This feature is very suitable for noise reduction in rotating machinery shock failure diagnosis. Sawalhi et al. [8] first used MED for rolling bearing and gear fault diagnosis. Taking advantage of the MED method with Spectral Kurtosis (SK) to further enhance the fault detection and diagnosis ability in the rolling bearing, Jiang et al. [9] detected the weak shocks of rolling element bearings using a MED method with envelope analysis. Wang et al. [10] extracted bearing failure frequency data successfully by MED and Ensemble Empirical Mode Decomposition (EEMD). As shown above, the MED method has demonstrated its power in rotating machinery fault diagnosis, but has not been put into use in multi-fault identification of rotating machinery.

In order to overcome the shortcomings of MED, McDonald et al. [11] proposed a rotating machinery fault feature extraction method, referred to as Multipoint Optimal Minimum Entropy Deconvolution Adjusted (MOMEDA). The method uses a time target vector to define the position and weight of the pulse sequence obtained by deconvolution. These targets are suitable for the feature extraction of a vibration source of a rotating machine that generates a shock pulse for every revolution. In addition, this method does not need an iterative algorithm to obtain the optimal filter. The MOMEDA algorithm can deal with non-integer numbers of fault periods and does not require re-sampling. In addition, the periodic components of the fault signal can be obtained by calculating the multi-point kurtosis of the vibration signal, which provides a new idea for the feature extraction of rotating machinery fault. However, MOMEDA as proposed by McDonald et al. focuses on the extraction of notch gear vibration signals which are strong shock signals. The main objective of this paper is to study multi-fault feature extraction in a strong noise environment with low Signal Noise Ratio (SNR). Through the simulation signals, Multipoint Kurtosis (MK) cannot accurately and effectively determine the multi-fault periods, resulting in the fact the MOMEDA method cannot extract the fault period signals based on the periods determined by MK, which requires original pre-filtered processed vibration signals. Therefore, in this paper, the noise reduction performances of MED at different SNRs are verified by the simulated signal, and the influence of strong noise on MK is also verified. Thus, the original vibration signal is denoised to improve the SNR, and then MK is introduced to extract the features of the fault period. Finally, by setting different period intervals, MOMEDA is used as the filter to extract the components of multiple faults, which can effectively identify the fault characteristics of wind turbine gearboxes.

## 2. Background and New Method 

### 2.1. Introduction of MED

Minimum entropy deconvolution (MED) first proposed by Wiggins, is an adaptive system identification method which was used by Sawalhi [11] for rolling bearing and gear fault diagnosis. The basic principle of MED is to solve the deconvolution results to highlight a few large spikes, which is a necessary pre-requisite for minimum entropy deconvolution. The maximum kurtosis is used as the iteration termination condition [7]. According to the maximum principle of kurtosis, a larger kurtosis value indicates a larger proportion of the shock component in the signal. This feature is suitable for noise reduction of rotating machinery shock failure signals, as it can better highlight shock pulses. We assume that the rolling bearing failure signal can be expressed as:

(1) y(n)=h(n)×x(n)+e(n)

where *e*(*n*) is the noise, *x*(*n*) is the shock sequence of the rolling bearing, *h*(*n*) is the transfer function, and *y*(*n*) is the collected vibration signal. 

The source signal characteristics will be lost after *x*(*n*) decays to *y*(*n*) due to the environmental noise and path transmission, resulting in a large entropy change. The purpose of solving for deconvolution is to obtain an inverse filter *f*(*n*) that recovers the property of the input *x*(*n*) according to the output, which is:

(2) x(n)=f(n)×y(n)

$\hat{f}\left(n\right)$ is an estimate of f(n), and its optimality is determined by the sequence $\hat{x}\left(n\right)$ obtained by solving deconvolution of Equation (2). The closer the shape of the sequence $\hat{x}\left(n\right)$ is to the shape of *x*(*n*), f(n) is optimal. This method is called minimum entropy deconvolution because the inverse filter $\hat{f}\left(n\right)$ can make $\hat{x}\left(n\right)$ restore the original property or have most of the original properties, that is, to minimize the entropy. Ralph Wiggins [7] uses the norm of sequence $\hat{x}\left(n\right)$ to determine the entropy of itself and uses it as the objective function to solve the optimal solution. When the sequence norm is the largest, the inverse filter $\hat{f}\left(n\right)$ is the optimal value, and Equation (2) becomes:

(3) $$x\left(n\right)=f\left(n\right)\times y\left(n\right)=\sum_{l=1}^{L}f\left(n\right)y\left(n-l\right)\sqrt{a^{2}+b^{2}}$$

According to the above analysis, the MED algorithm to find the minimum entropy can be summarized as follows:

(1) The elements in $f^{\left(0\right)}$ are all initialized to 1.
(2) The iterative calculation of the equation $x\left(n\right)=f{\left(n\right)}^{\left(i-1\right)}\times y\left(n\right)$ is performed.
(3) Calculate $b^{\left(i\right)}\left(l\right)=a\sum_{n=1}^{N}x^{3}\left(n\right)y\left(n-l\right)$.
(4) Calculate $f^{\left(i\right)}=A^{-1}b^{\left(i\right)}$.
(5) If ${\left\|f^{\left(i\right)}-f^{\left(i-1\right)}\right\|}_{2}^{2}$ is less than a given threshold, the threshold of this paper is 0.01, and recursion is stopped. Otherwise, let *i* increase by 1, and return to step 2.

### 2.2. Introduction of MOMEDA

Despite the successful results with MED, there are several major drawbacks. Firstly, MED is optimizing the norm kurtosis which prefers a solution of a single impulse. Secondly, MED is an iterative approach that involves iteratively finding a good filter solution [11]. In order to overcome the shortcomings of MED in the detection of rotating machinery faults, McDonald proposed in 2016 a position multi-pulse target recognition deconvolution algorithm with known positions for rotating machinery fault detection, which can identify continuous impulse pulses. The introduced maximum problem is called the multipoint optimal minimum entropy deconvolution adjusted (MOMEDA):

(4) $$(\vec{y},\vec{t})=\frac{1}{\left\|\vec{t}\right\|}\frac{{\vec{t}}^{T}\vec{y}}{\left\|\vec{y}\right\|}$$

(5) $$(\vec{y},\vec{t})=\underset{\vec{f}}{\max}\frac{{\vec{t}}^{T}\vec{y}}{\left\|\vec{y}\right\|}$$

where the target vector $\vec{t}$ is a constant vector that determines the pulse position and the weight. The normalized level 1 is used to denote the optimal target solution, and the fault period can be extracted at different sampling rates. The periods of different fault features at the same sampling frequency can also be identified. Therefore, the target vector can be used to separate the pulse signal and determine the position.

The extremum of Equation (5) is solved by deriving the filter coefficients ($\vec{f}=f_{1},f_{2},\ldots f_{L}$):

(6) $$\frac{d}{d\vec{f}}\left(\frac{{\vec{t}}^{T}\vec{y}}{\left\|\vec{y}\right\|}\right)=\frac{d}{d\vec{f}}\frac{t_{1}y_{1}}{\left\|\vec{y}\right\|}+\frac{d}{d\vec{f}}\frac{t_{2}y_{2}}{\left\|\vec{y}\right\|}+\ldots +\frac{d}{d\vec{f}}\frac{t_{N-L}y_{N-L}}{\left\|\vec{y}\right\|}$$

Since:

$$\frac{d}{d\vec{f}}\frac{t_{k}y_{k}}{\left\|\vec{y}\right\|}={\left\|\vec{y}\right\|}^{-1}t_{k}{\vec{M}}_{k}-{\left\|\vec{y}\right\|}^{-3}t_{k}y_{k}X_{0}\vec{y},\;\mathrm{and}\;{\vec{M}}_{k}=\left[\begin{array}{c} x_{k+L-1}\\ x_{k+L-2}\\ \vdots \\ x_{k} \end{array}\right]$$

Therefore Equation (6) can be written as:

(7) $$\frac{d}{d\vec{f}}\left(\frac{{\vec{t}}^{T}\vec{y}}{\left\|\vec{y}\right\|}\right)={\left\|\vec{y}\right\|}^{-1}\left(t_{1}{\vec{M}}_{1}+t_{2}{\vec{M}}_{2}+\ldots +t_{N-L}{\vec{M}}_{N-L}\right)-{\left\|\vec{y}\right\|}^{-3}{\vec{t}}^{T}\vec{y}X_{0}\vec{y}$$

Further simplified:

(8) $$t_{1}{\vec{M}}_{1}+t_{2}{\vec{M}}_{2}+\ldots +t_{N-L}{\vec{M}}_{N-L}=X_{0}\vec{t}$$

The extreme value is solved by the derivative equal to $\vec{0}$, and Equation (6) can be written as:

(9) $${\left\|\vec{y}\right\|}^{-1}X_{0}\vec{t}-{\left\|\vec{y}\right\|}^{-3}{\vec{t}}^{T}\vec{y}X_{0}\vec{y}=\vec{0}$$

That is:

(10) $$\frac{{\vec{t}}^{T}\vec{y}}{{\left\|\vec{y}\right\|}^{2}}X_{0}\vec{y}=X_{0}\vec{t}$$

Since $\vec{y}=X_{0}^{T}\vec{f}$, and assume that ${\left(X_{0}X_{0}^{T}\right)}^{-1}$ exists:

(11) $$\frac{{\vec{t}}^{T}\vec{y}}{{\left\|\vec{y}\right\|}^{2}}\vec{f}={\left(X_{0}X_{0}^{T}\right)}^{-1}X_{0}\vec{t}$$

The MOMEDA filter and the output are simplified as follows:

(12) $$F=\left[\begin{array}{cccc} {\vec{f}}_{1} & {\vec{f}}_{2} & \ldots & {\vec{f}}_{M} \end{array}\right]={\left(X_{0}X_{0}^{T}\right)}^{-1}X_{0}\left[\begin{array}{cccc} {\vec{t}}_{1} & {\vec{t}}_{2} & \ldots & {\vec{t}}_{M} \end{array}\right]$$

(13) $$Y=\left[\begin{array}{cccc} {\vec{y}}_{1} & {\vec{y}}_{2} & \ldots & {\vec{y}}_{M} \end{array}\right]X_{0}^{T}F$$

This method can completely avoid the iterative operation, and we do not need to consider whether the period is an integer and the influence of filter length on noise reduction.

MOMEDA can calculate several consecutive target vectors by Equations (12) and (13), and further distinguish between the fault periods and the relevant factors of its surrounding non-fault periods. However, when the noise is too large, the target vector t representing the position and weight of the output deconvolution impulse will be distorted, thus distorting the characteristics of the original periodic shock, as discussed in the next section of the simulation signal.

In order to extract the fault features accurately, Multipoint Kurtosis (MK) is introduced as a measure of feature extraction for the multi-stage transmission gearbox with wide frequency distribution and multi-fault period:

(14) $$\mathrm{Multipoint}\;\mathrm{Kurtosis}=\frac{\left(\sum_{n=1}^{N-L}t_{n}^{2}\right){}^{2}}{\sum_{n=1}^{N-L}t_{n}^{8}}\frac{\sum_{n=1}^{N-L}{\left(t_{n}y_{n}\right)}^{4}}{\left(\sum_{n=1}^{N-L}y_{n}^{2}\right){}^{2}}$$

This definition is based on kurtosis, but its target vector is extended to multiple pulses at the controlled position and further normalized to extract the fault period and plot the spectrum using multi-point kurtosis to identify the fault. When the multi-point kurtosis comes to a peak, the corresponding number of sampling points (period) is the fault period. In fact, there are peaks at integer, half, or 1.5 times the fault period, so multi-point kurtosis can differentiate between the fault periods and the surrounding non-fault periods.

## 3. Performance Evaluation by Simulated Signals

To observe the noise reduction performance of MED, a simulated signal is generated based on the impact characteristics of faulty bearings and rotors. It is then evaluated by applying MED to different cases when the signal is added with different levels of noise:

(15) $$signal\;1=1.8e^{-7.1\pi t}\sin\left(71\pi \sqrt{1-{\left(0.1\right)}^{2}t}\right)$$

(16) $$signal\;2=0.45e^{-4\pi t}\sin\left(40\pi \sqrt{1-{\left(0.1\right)}^{2}t}\right)$$

**Figure 1:**
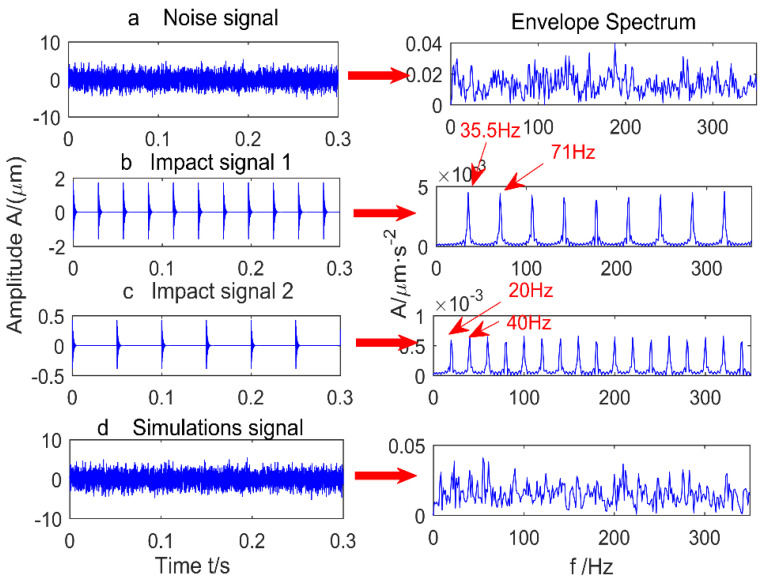
Simulation signal.

**Figure 2:**
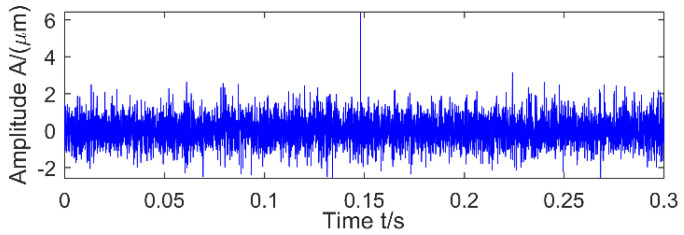
Using MED noise reduction.

**Figure 3:**
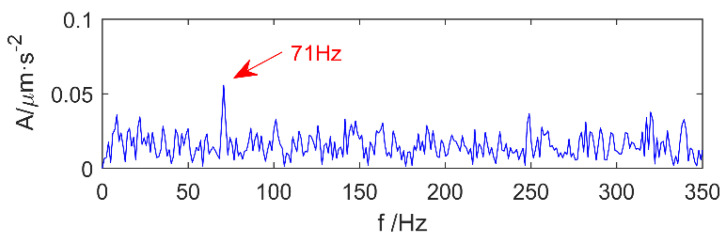
Envelope spectrum after MED noise reduction.

The time-domain signal shown in Figure 1d is a direct addition of three signals: Signal 1 representing a strong impulse-train with a shorter period, Signal 2 being a weak impulse-train with a larger period, and a random noise series. Envelope spectrum analysis of the simulation signal shows that under strong noise conditions, the shock signals are both submerged by noise, so it is necessary to reduce the noise. After filtering the simulation signal using MED, the kurtosis value increases from 1.5662 to 5.546. The results are shown in Figure 2, and there is only one peak, which cannot be determined as a strong or weak shock. The result of envelope spectrum analysis after MED noise reduction is shown in Figure 3, where the strong shock signals are extracted, but weak shock signals is still submerged by noise. Therefore, MED is only suitable for improving the signal-to-noise ratio of the vibration signal, and the tracking of the weak fault characteristic is somewhat difficult. 

### 3.1. MED Denoised MK Spectrum

**Figure 4:**
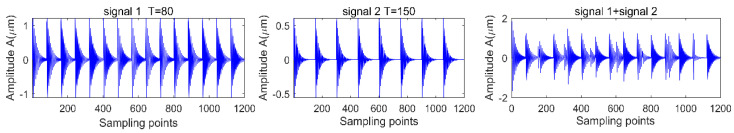
Simulation signals 1, 2 and 1 + 2.

In this part, the limitations of MK to extract the shock period at different SNRs are first verified. Further, the fault periods of the signals de-noised by MED are extracted with MK to verify its validity. The simulation signal is shown in Figure 4. 

**Figure 5:**
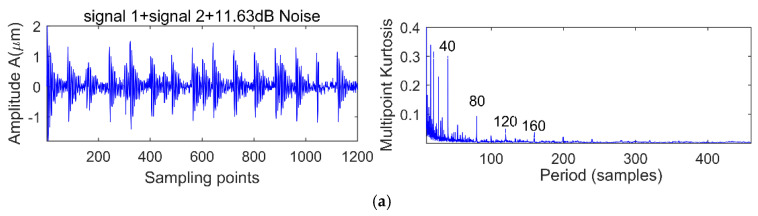

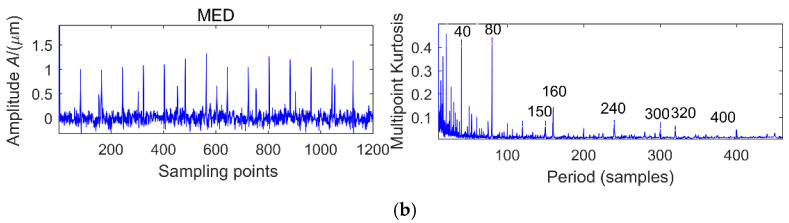
Simulation signal 1 + signal 2 + 11.63 dB noise before and after noise reduction analysis. (**a**) Simulation signal 1 + signal 2 + 11.63 dB noise and its multi-kurtosis (MK) spectrum; (**b**) Simulation signal 1 + signal 2 + 11.63 dB noise + MED and its MK spectrum.

**Figure 6:**
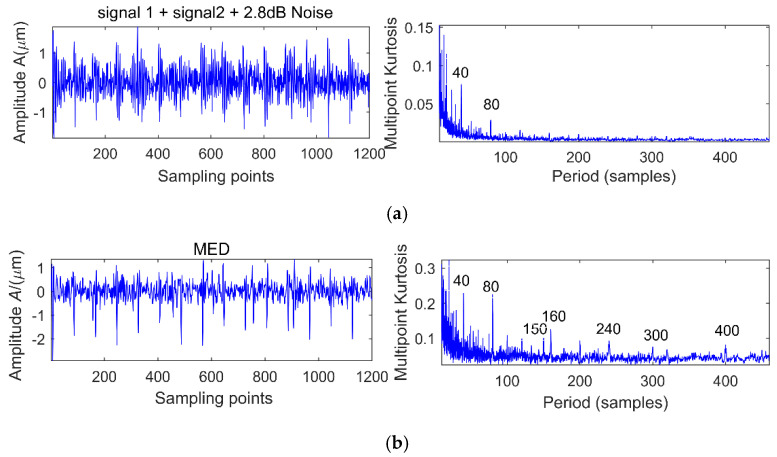
Simulation signal 1 + signal 2 + 2.8 dB noise before and after noise reduction analysis. (**a**) Simulation signal 1 + signal 2 + 2.8 dB noise and its MK spectrum; (**b**) Simulation signal 1 + signal 2 + 2.8 dB noise + MED and its MK spectrum.

**Figure 7:**
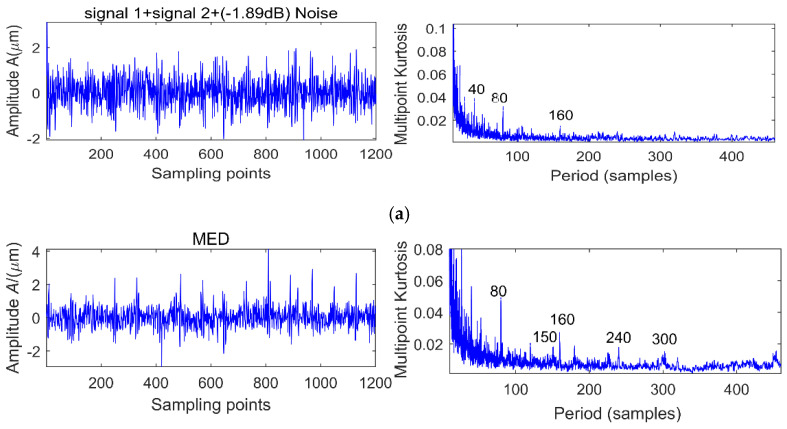
Simulation signal 1 + signal 2 + (−1.89 dB) noise before and after noise reduction analysis. (**a**) Simulation signal 1 + signal 2 + (−1.89 dB) noise and its MK spectrum; (**b**) Simulation signal 1 + signal 2 + (−1.89 dB) noise + MED and its MK spectrum.

There are two shocks and their period is 80 and 150 respectively, and the energy of signal 1 is greater than signal 2. We add different white noise levels to signal 1 and signal 2. The noise levels are 11.63, 2.8, −1.89 and −5.12 dB, respectively, and the MK spectra are shown in Figure 5a, Figure 6a and Figure 7a. It is clear that at the peak of the spectrum, the periods 40, 120, 80, 160, 240 correspond to 0.5 times, 1.5 times and integer multiples of the shock signal 1, respectively, but there is no spectrum peak about the period of 150, in addition, along with the increase of noise, the spectrum peak of period 80 is gradually submerged by noise when the noise level is −5.21 dB. 

In order to extract information about the period of 150, we use MED to reduce the noise of the original signal, and the corresponding multi-kurtosis spectra are shown in Figure 5b, Figure 6b and Figure 7b. It is clear that at the peak of the spectrum, the periods 75, 150 and 300 correspond to 0.5 times and an integral multiples of the shock signal 2, respectively.

### 3.2. MED Denoised MOMEDA

**Figure 8:**
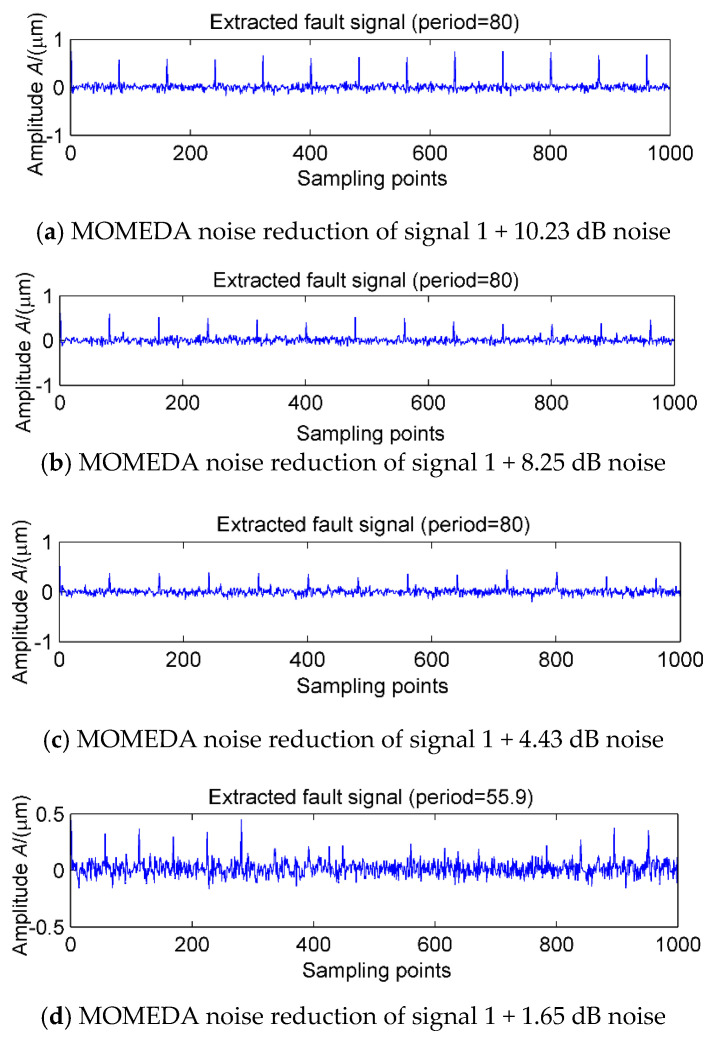

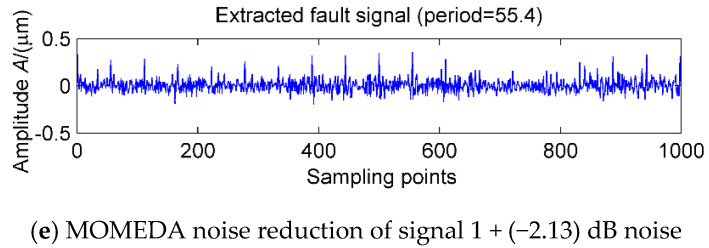
MOMEDA noise reduction of Simulation signal 1 + different white noise levels.

**Figure 9:**
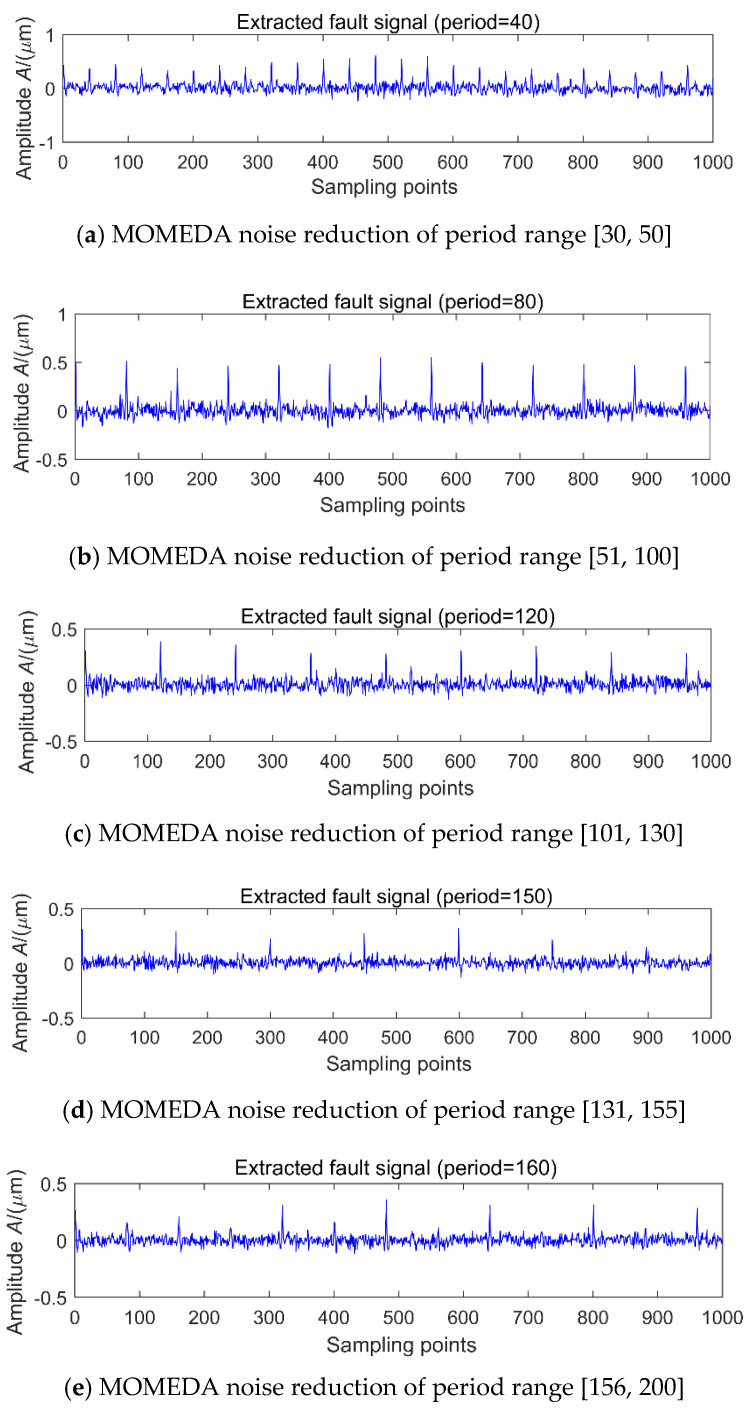
Signal 1 + signal 2 + 11.63 dB noise MOMEDA noise reduction of different period range.

In order to further verify the effect of noise on MOMEDA noise reduction proposed in the previous section, the simulation signals 1 in Figure 4 and different levels of noise are selected for analysis. Under the premise of selecting the noise reduction interval of [20, 160], the noise levels are 10.23, 8.25, 4.43, 1.56 and −2.13 dB, respectively, and the MOMEDA results are shown in Figure 8a–e. It is clear that with the decrease of the SNR, the periodic shock of 80 is gradually submerged, and with the increase of noise, MOMEDA can still extract a periodic shock (T = 55.4, 55.9) each time, as is shown in Figure 8d,e. However, this shock has no relationship with the signal of period 80. The main reason is that the target vector will be deformed in a strong noisy environment, which will distort the characteristics of the original periodic shock and further cause false diagnosis or leakage. If MOMEDA noise reduction is used in the case of multi-fault coexistence, it not only cannot extract the strong shock, but the extraction of the weak shocks is incapacitated. The next step will be to further discuss the noise reduction and set reasonable period intervals to improve the noise reduction efficiency of MOMEDA. In order to further extract the shock components corresponding to each fault period, we use MOMEDA to extract the features of the above simulated signal, but the range of the search cycle of this method is man-made. The signals in Figure 5a are denoised by MOMEDA by setting up the different period ranges of [30, 50], [51, 100], [101, 130], [131, 155] and [156, 200], and the results are shown in Figure 9a–e. 

It is clear that under low noise conditions, a unique peak can be highlighted in each cycle interval, but the extracted peak may be 0.5 times (Figure 9a), 1.5 times (Figure 9c) and 2 times (Figure 9e) the original signal period (Figure 9b,d). The half cycle 75 of the original shock signal 2 is not highlighted in the [51, 100] range, since the main energy in this interval is concentrated in the original shock signal 1. In addition, when the period interval is set to [20, 200], only the shock signal with period 80 can be extracted. The main reason is that when there are two fault periods in the composite signals, and the signal with period 80 is stronger than that of 150, so when using MOMEDA to extract the periodic component, the shock component with a period of 80 is successfully identified, but the shock component with a period of 150 is always mistaken for noise being filtered out. Therefore, when the multi-fault features are extracted, the setting of the fault period intervals will affect the precision of fault extraction. 

**Figure 10:**
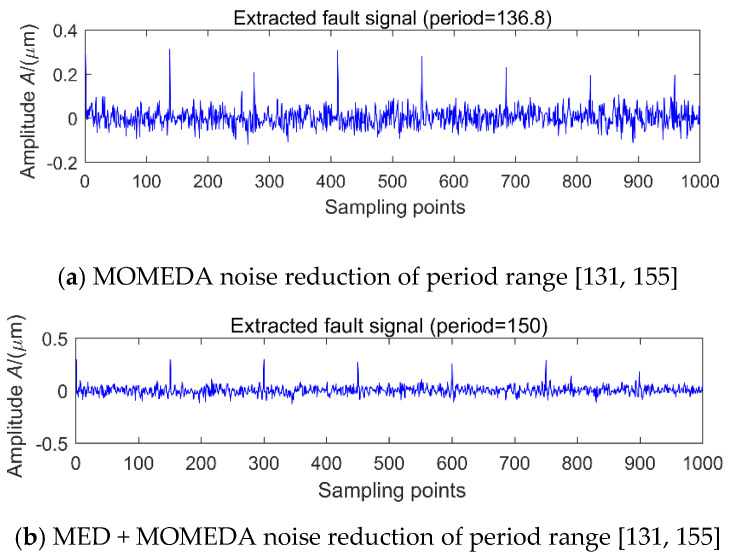
Signal 1 + signal 2 + (−1.89 dB) noise reduction analysis of period range [131, 155].

When the SNR is low, by entering the different period interval of [30, 50], [51, 100], [101, 130], [131, 155] and [156, 200], the simulated signal shown in Figure 7a is denoised by with MOMEDA. Due to the period of 80 signal is stronger, so its MOMEDA noise reduction is similar to Figure 9, but the results of the noise reduction of the [131, 155] interval are shown in Figure 10a, the corresponding period of 136.8 is a pseudo-periodic component, which has no relationship with the simulated signals of the original periods 80 and 150. This means that in a strong noisy environment, the search results in the interval of a fault period may be noise, which may lead to misdiagnosis. In order to eliminate noise interference, the original signal is first denoised by MED and then extract the period, so that the noise interference can be successfully eliminated, the results is shown in Figure 10b.

Therefore, a target that has strong and weak faults can be simultaneously extracted by MED noise reduction and different period interval setting. For example, when the periodic signals 80 and 150 are searched, the search interval may be set to [70, 90] and [140, 170], then the periods corresponding to other faults or noises are assigned to the noise components.

## 4. Multi-Fault Feature Recognition under Strong Noise

Taking the following aspects into account:

(1) When the background noise of the gearbox is large, the fault features are often submerged by noise and the fault period is not easy to be extracted. Therefore, by simulation signal of Section 3, it can be found that multi-kurtosis cannot identify the fault features in the case of a single fault or multiple faults at low signal-to-noise ratio as shown in Figure 8a. We can see that the peaks at the period of 80 and 150 are not obvious, and there is no peak at the multiple or half times. Thus, a strong noise reduction method is urgently needed for its pretreatment.

(2) In order to extract the characteristic period of multiple faults, we can improve the signal-to-noise ratio of the signal through the MED pre-noise reduction method. Because the maximum kurtosis is taken as the iteration termination condition in MED, it not only can highlight the individual strong shock signals, but also improve the signal-to-noise ratio of the weak shock signals. Then we multiply the kurtosis of the signal after noise reduction. It can be seen from Figure 5b, Figure 6b, Figure 7b and Figure 8b that the positions at which peaks appear are at periods 40, 80, 160 and 150, 300, respectively. Obviously, the signal after noise reduction, although not as large as the peak corresponding to Figure 5b, has distinguished the fault period and the surrounding non-fault period effectively. Therefore, for a periodic vibration signal, when the multi-kurtosis is after MED it has a fixed period corresponding to the peak period and its multiples.

The fault period and reasonable search intervals are determined firstly, and then the signals de-noised by MED are processed with MOMEDA to obtain the fault features. Since each feature extraction can extract only one periodic component, when using MOMEDA to extract each period component, if the period range is set too large, other noise components will be extracted, resulting in misdiagnosis. In this case, in order to improve the accuracy of MOMEDA search fault period, the fault period should be adjusted. 

**Figure 11:**
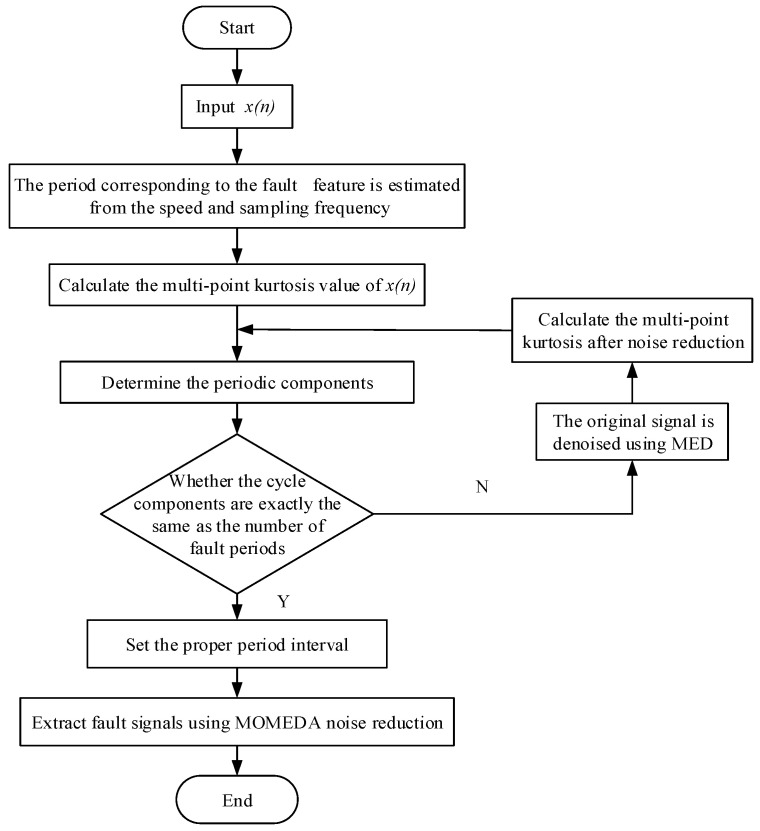
Feature extraction flow chart.

In order to avoid leakage diagnosis in the process of noise reduction by MOMEDA, the scope of the fault period needs to be identified in advance to further determine the number of sampling points. In order to make sure that there will be more than five period peaks in the whole sampling interval, the fault period of the gear box rotating machinery should be determined, and the sampling points should be controlled at about five times the maximum period. The specific feature extraction flow chart is shown in Figure 11.

## 5. Application Case

**Figure 12:**
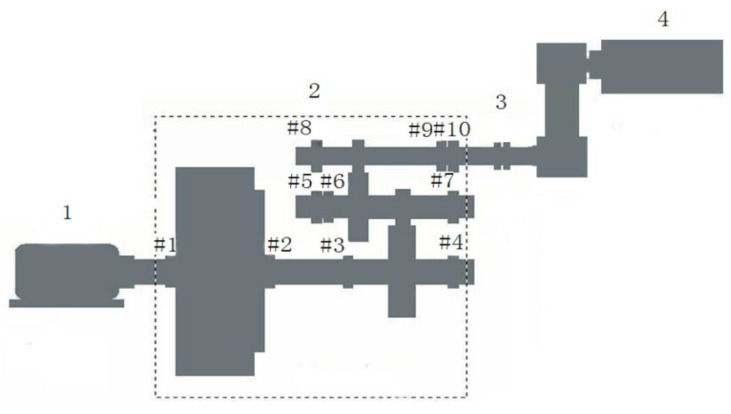
Schematic of wind turbine gearbox test rig.

The method is further demonstrated with a vibration signal obtained from a wind turbine. The test bench sketch of the wind turbine gearbox is shown in Figure 12. The gear transmission system has three stages. The first is a planetary gear train and the other two are the fixed axis gear trains. According to the structural features of the wind turbine gearbox test bench, four transducers are mounted at the measuring points on bearing blocks #5, #7, #8, and #10, respectively. The rotational frequency of the output shaft is 27.3 Hz. The sampling frequency for data acquisition is at 10,000 Hz. According to the calculation, the vibration period of the shaft is 365.9. In addition, according to the bearing type and the shaft speed to calculate the bearing failure frequency and fault period, and bearing fault periods are also listed in the following Table 1.

**Figure 13:**
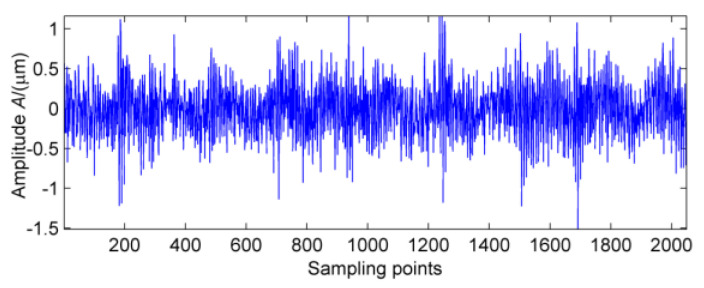
No-load vibration signal.

**Figure 14:**
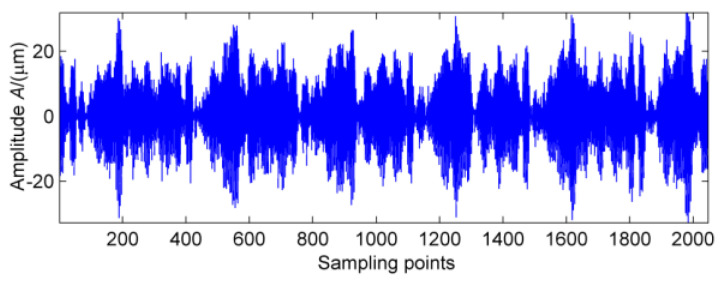
Vibration signal under strong load.

**Figure 15:**
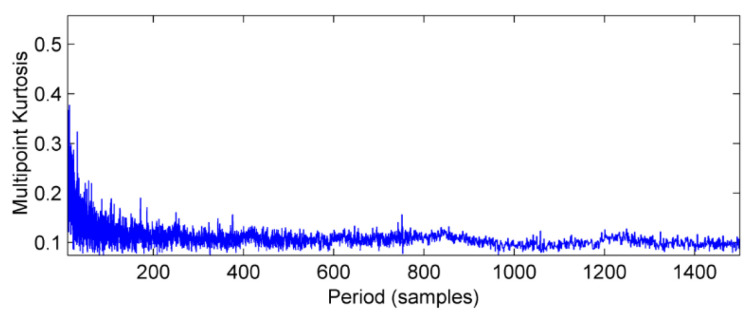
Multi-point kurtosis spectrum of vibration signal under no-load condition.

**Figure 16:**
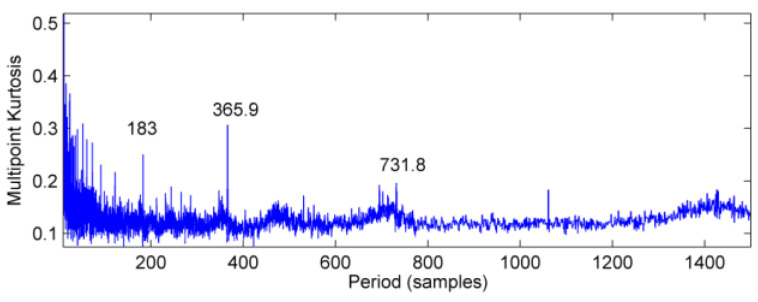
Multi-point kurtosis spectrum of vibration signal under strong load.

The vibration signal data of bearing block #10 under no-load and strong load conditions are collected, respectively, and the vibration wave forms are shown in Figure 13 and Figure 14. In Figure 13, there is no obvious periodic component, the vibration amplitude is small, and the vibration signal is stable. When the power from the generator is increased to 1880 kW, vibration and noise begin to increase gradually. At the same time, there are obvious signs of warming in the high speed shaft of the gear box after the test rig running for a short time. It can be seen from Figure 14 that there is an obvious periodic component. In order to further determine the failure period, the multi-point kurtosis of the fault signals under no-load and strong load conditions were calculated. According to the bearing fault period and the rotation period of the shaft, set the multi-point kurtosis period range of 10~1500, step size is set to 0.1, and the results are shown in Figure 15 and Figure 16.

**Figure 17:**
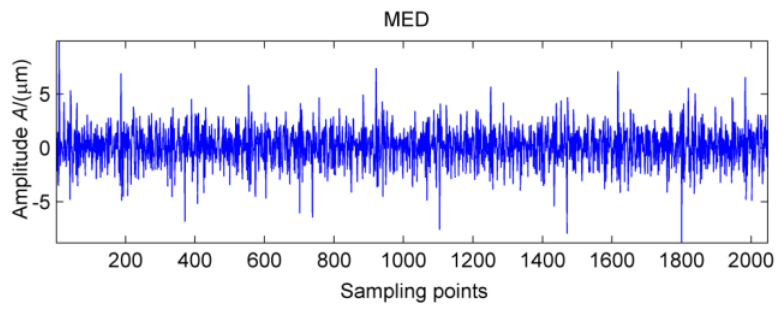
MED reduction of vibration signal under strong load.

Obviously, the multi-point kurtosis spectrum of the normal gearbox is smooth, but the 183 Hz, 365.9 Hz, 731.8 Hz periodic components in the fault spectrum correspond to the multiple rotation period of the high-speed shaft. As a result, it can be preliminarily determined that the high-speed shaft has undergone weak bending. As the noise is relatively large, in order to determine whether there are other fault components in the gearbox, according to the method described in this paper, using the MED method to reduce the noise of the vibration signal processing to get the signal after noise reduction which is shown in Figure 17. It can be seen that kurtosis value has been greatly improved, but no significant periodic weak shock signal appears. 

**Figure 18:**
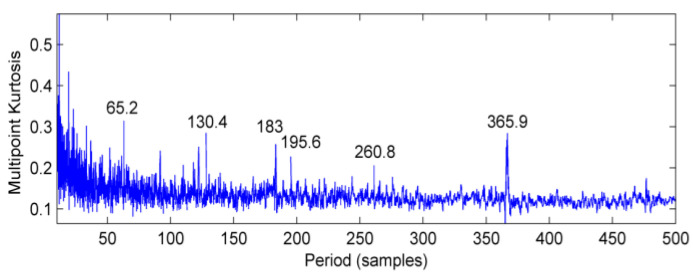
Multi-point kurtosis spectrum of the signal after MED noise reduction.

The de-noised signals are analyzed by multi-point kurtosis. At this time, the problem of bending failure of the high-speed shaft has been determined. Therefore, the period range is set to be 10~500, the step is 0.1, and the result is shown in Figure 18. Except for the 183 Hz and 365.9 Hz high-speed shaft period components, the spectrum peak also appear in the 65.2 Hz, 130.4 Hz, 195.6 Hz, 260.8 Hz components. The observation of Table 1 shows that these frequency components are multiple of the fault frequency of outer ring of #10 bearing. Thus, it can be determined that the failure of the gearbox consists mainly of two aspects, besides high-speed shaft bending, the outer ring of output bearing has also failed.

**Figure 19:**
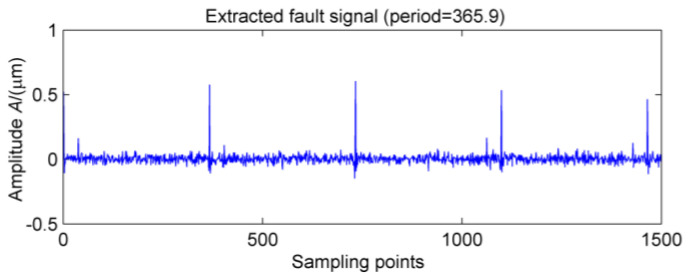
MOMEDA of the signal after MED noise reduction.

**Figure 20:**
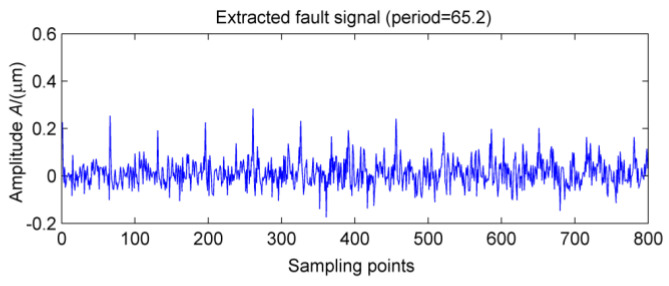
MOMEDA of the signal after MED noise reduction.

In order to further extract the fault signal, the original signals are denoised by MOMEDA. The two period ranges are set of 360~370 and 60~70, respectively. The corresponding spectra of the extracted fault features are shown in Figure 19 and Figure 20, respectively. Obviously, the shock vibration signals are extracted one by one, and when the box is finally opened, it could found that outer ring of #10 bearing displays a serious pitting phenomenon.

**Figure 21:**
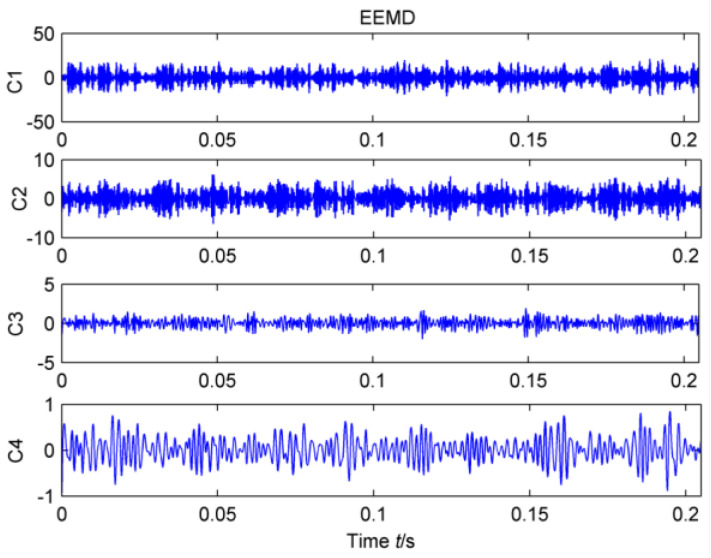
Noise reduction with EEMD.

**Figure 22:**
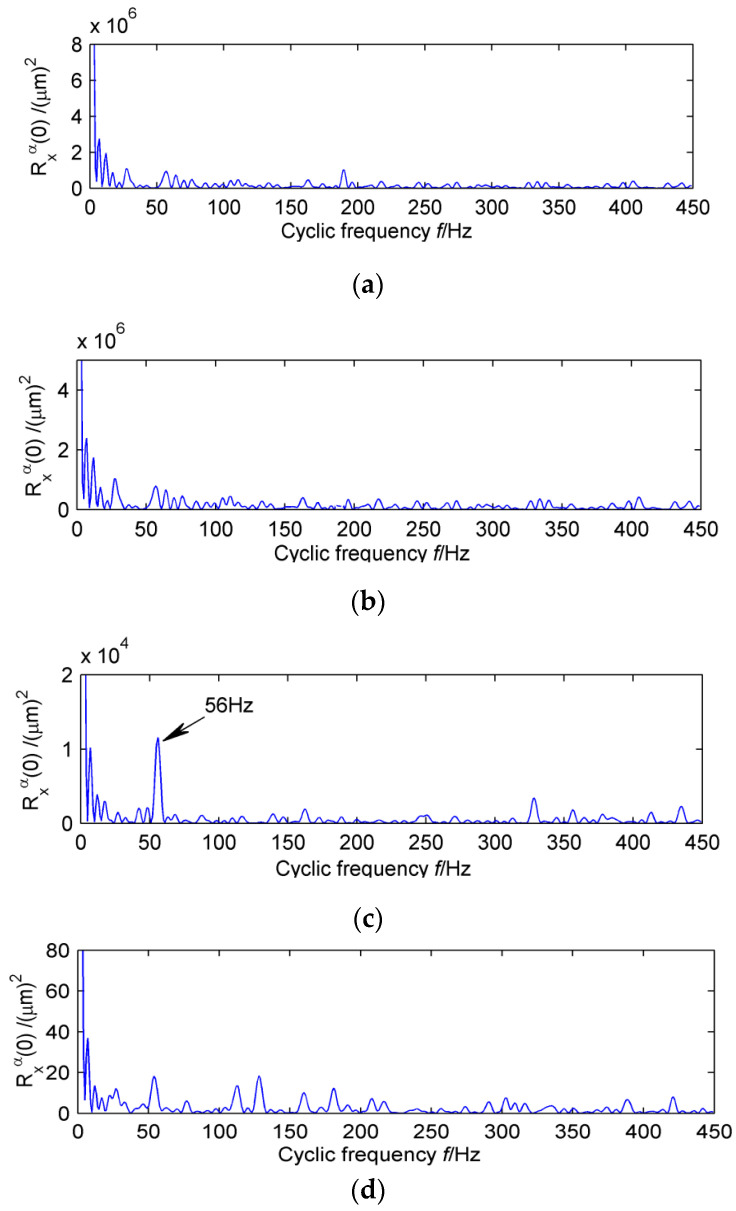
The first four IMFs with cyclic autocorrelation function.

In order to verify the validity of the proposed method, it can be compared with the traditional adaptive noise reduction method EEMD. In the case of white noise amplitude of 0.2, the original vibration signal is decomposed adaptively. The first four mode functions with the strongest correlations with the original signal are shown in Figure 21. The periodic component cannot be extracted accurately by the time-domain image. Therefore, the cyclic autocorrelation function is selected to perform demodulation analysis on each layer, and the result is shown in Figure 22. 

Obviously, only the intrinsic mode function of the third layer extracts the double frequency of the diagnosis frequency of the high-speed shaft. However, the failure frequency of the high-speed shaft #10 bearing is not successfully extracted in high frequency. It is possible that the weak bearing fault is still submerged by EEMD decomposition due to the background noise. Therefore, the EEMD cannot extract the composite fault features effectively.

## 6. Conclusions

(1) MED denoising using the maximum kurtosis as the termination condition of iteration can improve the SNR of the signals. Its primary advantage is that it can greatly improve the SNR of the original signal to prepare for the signal post-processing without changing the fault structure of the original signal. However, the limitation of this algorithm is that by only reaching the maximum kurtosis and finding the best filter, the iterative calculation can be stopped. In addition, this algorithm can only extract the strong shock components of individual periods, and has no immunity to weak shock signals. 

(2) In this paper, the multi-point kurtosis method was used to extract the fault-period components, the maximum multipoint period or the multiple of the period is the fault characteristic. This period component can be integer or fractional, without a priori knowledge. The effect of tracking the fault period through the maximum of multi-point kurtosis is relatively weak under strong background noise. The pre-processing of the original signal by MED can reduce the interference of the noise to the multi-point kurtosis method in extracting the periodic components. In addition, after determining the periodic components, it is necessary to set a reasonable period to extract the fault characteristics. Larger periodic intervals often contain some uncertain factors, such as noise components, and will further interfere with feature extraction.

(3) The validity of this method is proved by the agreement of the simulation signals and measured signals. Using this method, the fault characteristic of the composite fault can be successfully extracted, and it is effective even under strong background noise.

## Figures and Tables

**Table 1:** Bearing fault period data.

Bearing Locations	Inner Ring Fault Period	Outer Ring Fault Period	Rolling Element Fault Period
High speed input shaft bearing #8	42.3	59.9	100.4
High speed input shaft bearing #9	42.3	59.9	100.4
High speed output shaft bearing #10	45.1	65.5	108.3
